# Utility of a novel lipoarabinomannan assay for the diagnosis of tuberculous meningitis in a resource-poor high-HIV prevalence setting

**DOI:** 10.1186/1743-8454-6-13

**Published:** 2009-11-02

**Authors:** Vinod B Patel, Ahmed I Bhigjee, Hoosain F Paruk, Ravesh Singh, Richard Meldau, Cathy Connolly, Thumbi Ndung'u, Keertan Dheda

**Affiliations:** 1 Department of Neurology, University of KwaZulu-Natal, South Africa; 2 Hasso Plattner Research Laboratory, Doris Duke Medical Research Institute, Nelson R Mandela School of Medicine, University of KwaZulu-Natal, South Africa; 3 Lung Infection and Immunity Unit, Division of Pulmonology & UCT Lung Institute, Department of Medicine, University of Cape Town, South Africa; 4 Department of Infection, Centre for Infectious Diseases and International Health, University College London, London, UK; 5 Institute of Infectious Disease and Molecular Medicine, University of Cape Town, Cape Town, South Africa; 6 Biostatistics Unit, Medical Research Council, Ridge Road, Durban, South Africa

## Abstract

**Background:**

In Africa, tuberculous meningitis (TBM) is an important opportunistic infection in HIV-positive patients. Current diagnostic tools for TBM perform sub-optimally. In particular, the rapid diagnosis of TBM is challenging because smear microscopy has a low yield and PCR is not widely available in resource-poor settings.

**Methods:**

We evaluated the performance outcome of a novel standardized lipoarabinomannan (LAM) antigen-detection assay, using archived cerebrospinal fluid samples, in 50 African TBM suspects of whom 68% were HIV-positive.

**Results:**

Of the 50 participants 14, 23 and 13 patients had definite, probable and non-TBM, respectively. In the non-TB group there were 5 HIV positive patients who were lost to follow-up and in whom concomitant infection with *Mycobacterium tuberculosis *could not be definitively excluded. The test sensitivities and specificities were as follows: LAM assay 64% and 69% (cut-point 0.22), smear microscopy 0% and 100% and PCR 93% and 77%, respectively.

**Conclusion:**

In this preliminary proof-of-concept study, a rapid diagnosis of TBM could be achieved using LAM antigen detection. Although specificity was sub-optimal, the estimates provided here may be unreliable because of a classification bias inherent in the study design where it was not possible to exclude TBM in the presumed non-TBM cases owing to a lack of clinical follow-up. As PCR is largely unavailable, the LAM assay may well prove to be a useful adjunct for the rapid diagnosis of TBM in high HIV-incidence settings. These preliminary results justify further enquiry and prospective studies are now required to definitively establish the place of this technology for the diagnosis of TBM.

## Background

Tuberculosis is increasing in Africa [1], where HIV infection has fuelled an increasing prevalence of pulmonary and extra-pulmonary tuberculosis (TB) including tuberculous meningitis (TBM) [2,3]. In HIV-endemic settings, a common clinical dilemma in patients with neurological symptoms and cerebrospinal fluid (CSF) abnormalities, even when an alternative diagnosis is made, is whether the patient has tuberculosis. Biochemistry and cell counts are unreliable in HIV+ve patients, PCR is not widely available, smear microscopy of the CSF has a poor sensitivity (~5%) and culture results are delayed for several weeks [4]. Thus, the diagnosis of TBM, which is associated with substantial morbidity and mortality, is challenging in high HIV-incidence settings where current tools perform poorly. There is an urgent need to find alternative rapid ways to diagnose TBM. Although PCR is a useful rule-in test (60% sensitivity and 98% specificity); it is expensive, technically demanding and it not widely available in resource-poor settings. Alternative methods such as liquid-based culture provide results only after several weeks [5-7] and gas chromatography for tuberculostearic acid is expensive and has limited availability even in resource-rich settings [8]. The utility of quantitative antigen-specific T cell responses though recently described [9] has not been validated in clinical trials and is untested in TBM.

Lipoarabinomannan (LAM) is a glycolipid forming part of the mycobacterial cell wall. It has several immunomodulatory effects including interference with macrophage activation and antigen processing [10-13]. Serum LAM antibody responses have previously been evaluated as a diagnostic test for tuberculosis [14]. The performance outcomes of several other mycobacterial antigen and antibody detection kits have been variable, with sensitivities of 60 to 90% [14-19]. Zhang *et al *evaluated serum LAM antigen in patients with extra-pulmonary tuberculosis, including three patients with TBM, and reported a sensitivity of 26.7% in the extra-pulmonary tuberculosis group [20]. More recently, a novel standardized ELISA-based assay was developed to detect LAM antigen in urine [21-23]. Significantly, a prototype point-of-care immuno-chromatographic strip test format is now in clinical trials using urine, sputum and saliva. However, the commercially available LAM antigen-detection assay has not previously been evaluated in CSF. To investigate the possible utility of this novel technology for the diagnosis of TBM we performed a preliminary study using archived CSF samples from 50 TBM suspects [24].

## Methods

### Patients

Following ethical approval from the Biomedical Research Ethics Administration of the University of Kwazulu-Natal, (consent from patients was not obtained for this retrospective study), LAM antigen levels were measured in CSF samples obtained by lumbar puncture, stored for the past three years at -70°C, from 50 consecutively-recruited untreated TBM suspects referred to a tertiary institution in Durban, South Africa between January 2004 and December 2005. The culture, PCR and microscopy tests were performed on the fresh samples at the time of recruitment, while the LAM detection was done on stored frozen samples. The microbiological results have been described in a previous publication [24]. Approximately 30% of patients referred to our unit per annum (686 admissions for year 2005) have neurological tuberculosis and 80% of these are HIV positive. CD4 counts were not available at our centre at the time of HIV testing. After diagnostic work-up and re-review of patient notes, and follow-up data, 50 patients were classified as:

**(1) **Definite TBM if the CSF culture was positive for *M. tuberculosis *(the gold standard).

**(2) **Probable TBM if the clinical, CSF and radiological findings were consistent with TBM, but the culture was negative and alternate aetiologies were excluded. All patients in this category received empiric anti-TB treatment.

**(3) **Non-TB if another aetiology was found to explain the clinical presentation and no anti-TB treatment was administered. However, in five HIV positive patients, no follow-up was available and thus the concomitant presence of *M. tuberculosis *infection could not be excluded.

### CSF testing

The following tests were applied to all CSF samples to rule-in or exclude other diseases: real-time PCR for *M. tuberculosis *[24], antigen-detection test for cryptococcus, serology for syphilis and cysticercosis, and PCR for herpes viruses (Herpes Simplex virus 1 & 2, cytomegalovirus, CMV, Varicella Zoster virus).

LAM antigen was measured using an ELISA kit (Clearview^® ^TB ELISA, Inverness Medical Innovations, USA). The samples were thawed and allowed to equilibrate to room temperature. After an initial heating step to separate antigen-antibody complexes, CSF samples were seeded, in duplicate, into 96 well plates coated with anti-LAM antibodies. Following this an ELISA was done to measure optical density (OD) determined by a technician blinded to patient details. The LAM concentrations were extrapolated from a standard curve constructed from two-fold serial dilutions (8 in total ranging from 10 to 0.08 ng/ml) of the LAM antigen (20 ng/ml), supplied by the manufacturer. To evaluate the clinical utility of the new test, a comparative clinical predictive score was applied as defined by Thwaites *et al *[25] using age, duration of symptoms, total blood white cell count, percentage neutrophils in CSF and total cell count in CSF, from which a score was derived which if <4 predicted for TBM.

### Data analysis

Statistical analysis was conducted using STATA-10. In the definite TBM group (1), for the sensitivity calculation, the number of culture positive samples (n = 14) served as the denominator and for specificity calculation the number of non-TB samples (n = 13) served as the denominator. In the probable TBM group (2), the number of probable TBM cases (n = 23) served as the denominator for the sensitivity calculation. For the specificity calculation, the number of non-TBM samples (n = 13) served as the denominator. The manufacturer-recommended cut-off point for urine samples was used: if the OD was > 0.1 above the OD of the negative control, the patient sample was regarded as being positive. An additional analysis was conducted using area under the receiver operating curve (ROC) derived with the OD cut-off point > 0.22 above the negative control for a positive test result. Performance data were re-derived using this cut-off point.

## Results

All the patients were black African (mean age of 30.3 years; 60% female; 34/50 (68%) were HIV positive, eight patients HIV (16%) status was unknown and eight patients were HIV negative (16%). All except three patients had AIDS defining illnesses such as extra-pulmonary tuberculosis, cryptococcal meningitis, cerebral toxoplasmosis, CMV encephalopathy or disseminated herpes zoster. Of the 50 participants, there were 14 patients in the definite group (1), 23 in the probable group (2) and 13 patients in the non TBM group (3). The clinical changes in the CSF for the three patient groups are presented in Table 1. The 13 non-TBM patients (group 3) had the following diagnoses: cryptococcal meningitis (n = 4), cerebral toxoplasmosis (n = 2), viral meningitis (n = 5: CMV = 2, herpes = 1, HIV = 2), 1 acute demyelinating encephalomyelitis and 1 epilepsy.

**Table 1:** Clinical characteristics of the cerebrospinal fluid in the definite, probable and non-tuberculous meningitis (TBM) groups.

**Diagnosis**	**Protein (g/l)**	**Lymphocyte count (cells/ml)**	**Neutrophil count (cells/ml)**	**Glucose (mmol/l)**
	**Mean (SD)**	**Mean (SD)**	**Mean (SD)**	**Mean (SD)**

**Definite TBM (14)**	1.8 (1.58)	64.3 (101.50)	74.3 (135.56)	**1.4 (0.69)**

**Probable TBM (23)**	1.5 (1.67)	32.4 (54.67)	123.7 (285.4)	**2.9 (1.08)**

**Non-TBM (13)**	0.9 (1.12)	66.0 (165.34	12.6 (16.4)	**2.9 (1.01)**

**Total (50)**	**1.4 (1.52)**	**50.1 (105.08)**	**81.0 (208.86)**	**2.4 (1.17)**

Of the 14 culture-positive samples (group 1), 13 were also positive by real-time PCR. A further 14 samples were PCR positive for *M. tuberculosis *DNA, but culture negative. Of these, 11 were in the TBM probable group and three were in the non-TBM group. None of the samples were smear positive, the test performance outcomes were similar in HIV positive and HIV negative groups, and there was no correlation between LAM antigen levels and CSF biochemical or cellular parameters (data not shown). The definite TB group (n = 14) consisted of nine LAM antigen positive and five LAM negative CSF samples. The probable TBM group (n = 23) consisted of six LAM positive and 17 LAM negative samples. In the non-TB group (n = 13) there were five LAM positive and eight negative (Table 2).

**Table 2:** Numbers of patients positive and negative for lipoarabinomannan (LAM) in the CSF in the definite, probable and non-tuberculous meningitis (TBM) groups

**Diagnosis (Number)**	**LAM +ve (%)**	LAM -ve (%)
**Definite TBM (14)**	9 (64)	**5 (36)**

**Probable TBM (23)**	6 (26)	**17 (74)**

**Non-TBM (13)**	**5 (38.5)**	**8 (61.5)**

The PCR sensitivities and specificities for the LAM antigen assay for groups 1 and 2 are shown in Table 3. In the definite TBM group, the sensitivity and specificity of the LAM antigen testing was 64% and 69% (when using the receiver operating characteristic (ROC) defined cut-point) respectively whereas the corresponding figures for the PCR assay were 93% and 77% respectively. The smear microscopy when 3 organisms were considered positive, was 0.0. A ROC curve was derived comparing definite TBM and non-TBM groups (Figure 1). When the sensitivity of the manufacturers and the study-derived OD cut-off points were compared, there was no difference (64% using both 0.1 and 0.22 OD cut-off points). The specificity increased from 62% to 69%.

**Table 3:** Sensitivity, specificity, positive predictive value (PPV) and negative predictive value (NPV) values for PCR test and lipoarabinomannan (LAM) ELISA assay in patients with definite and probable tuberculous meningitis (TBM).

		**Definite TBM (culture +ve), n = 14**		**Probable TBM, n = 23**	
		
		**percentage**	**95% CI**		**95% CI**
PCR	Sens	93	64 - 95	48	27 - 69
	
	Spec	77	46 - 94	77	46 - 94
	
	PPV	81	54 - 95	79	49 - 94
	
	NPV	91	57 - 100	46	25 - 67

LAM ELISA using 0.1 cut-off point^#^	Sens	64	36 - 86	26	11 - 49
	
	Spec	62	32 - 85	62	32 - 85
	
	PPV	64	33 - 85	55	16 - 54
	
	NPV	62	36 - 86	32	25 - 82

LAM ELISA using AUC-derived 0.22 cut-off point^$^	Sens	64	36 - 86	22	08 - 44
	
	Spec	69	39 - 90	69	39 - 90
	
	PPV	69	39 - 90	56	23 - 85
	
	NPV	64	36 - 86	33	17 - 54
	
	AUC	0.60	n/a	0.70	n/a

# the manufacturer-recommended cut-off point for testing of urine samples, 0.1; $ the area under the receiver operating curve-derived cut-point, 0.22.

**Figure 1:**
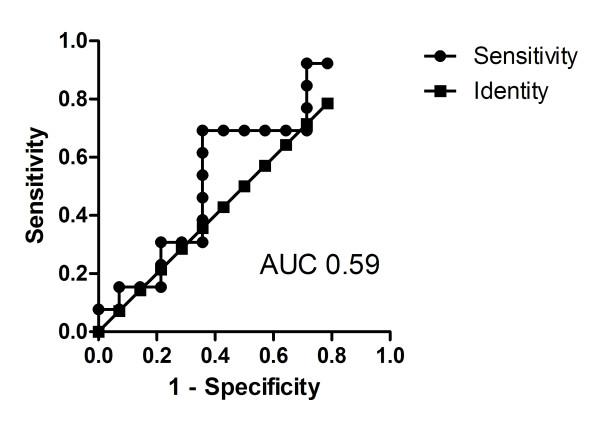
**Receiver operating characteristic curve (ROC) of the lipoarabinomannan (LAM) ELISA assay derived by comparing definite tuberculous meningitis (TBM) and non TBM groups**.

## Discussion

The LAM antigen-detection test, a potential test for TBM, is a rapid and relatively simple assay. In resource-poor settings, the availability of PCR is extremely limited, and thus smear microscopy for *M. tuberculosis *is the only rapid way to diagnose TBM. There have been several recent reports evaluating urinary LAM antigen as a diagnostic test for the diagnosis of pulmonary tuberculosis reporting sensitivities in the region of 60% and specificities of 88 to 96% [21-23,26]. In our study, compared to smear microscopy, which identified none of the TBM cases, the LAM ELISA detected nine out of 14 definite TBM cases. We speculate that the five undetected cases may have been due to paucibacillary disease, non-specific protein binding of antibody, or failure to separate CSF antigen-antibody complexes despite an intermediate heating step. However, significantly, the specificity was only 64% (five LAM+ve cases in the non-TB group). How do we explain these results? Four out of five of these cases were HIV positive and as the patients had been immediately discharged to remote referral centres after their diagnostic work-up no follow-up data was available. Thus, we cannot be sure that some or all of these patients had dual infection (identified alternative aetiology and concomitant early TBM), which is well-recognised in HIV positive patients with meningitis [27,28]. It is also possible that LAM antibody may have cross-reacted with mannose residues found in other organisms including *cryptoccus neoformans*. (1 out of 5 positive LAM assays in the non-TB group had cryptococcal meningitis) [29]. Thus classification bias rather than poor test performance could have accounted for the sub-optimal test specificity. Given the retrospective nature of the study it is impossible to determine which of these possibilities is correct and it is entirely possible that the test may lack sufficient specificity to be clinically useful. Thus, a prospective study is now required to clarify the true specificity of the LAM assay, paying careful attention to case definition and classification.

Several factors may modulate the level and hence detection of LAM antigen in biological samples. The influence of the blood brain barrier (BBB) permeability on LAM sensitivity in CSF is uncertain and has not been previously investigated. The frequency of LAM antigenemia or a measure of the IgG-albumin index could potentially have given us an idea about BBB function. However, this was not possible given the retrospective study design. The widely variable urine LAM sensitivity (17.8% to 80.3%) reported in several publications may be related to the severity of immune suppression in HIV-positive patients [22,23,26,30]. This aspect deserves further investigation in future studies.

It is possible that the LAM ELISA will have clinical utility in a resource-poor setting because it has incremental value over and above that of simple clinical and laboratory parameters, including a previously published bio-clinical score. Indeed, the clinical score (Thwaites et al) [25], devised primarily to distinguish bacterial meningitis from TBM, had a specificity of only 10% for the diagnosis of TBM. The rapidity of the LAM test (approximately 2 hours), despite a sensitivity of only 64%, makes it a potentially useful rule-in test in high-burden settings. Here the diagnosis is largely clinical and the CSF picture may be atypical, and even acellular, in HIV positive individuals [4,24].

## Conclusion

This preliminary data obtained through analysis of a small number of archived samples indicate that despite its modest sensitivity, the LAM assay, with a 60% positive predictive value may be promising for the rapid diagnosis of TBM in a resource-poor, high-HIV prevalence tertiary setting. Prospective trials are now warranted in different geographical and clinical settings to clarify the utility and specificity of this assay for the diagnosis of TBM.

## Competing interests

The authors declare that they have no competing interests.

## Authors' contributions

AIB and HFP assessed and collated samples. VBP drafted the manuscript. KD supervised, facilitated the laboratory processing and co-wrote the paper. TN supervised laboratory processing of samples, helped analyze and interpret laboratory data and provided laboratory resources. RS and RM provided the laboratory expertise for sample processing. CC provided statistical assistance. All authors have read and approved the manuscript.

## Author's information

K Dheda current address: Department of Medicine. J floor, Old Main Building, Groote Schuur Hospital, Observatory, Cape Town, 8000, South Africa.
